# Supplementary material for: Gene-specific transcriptional activation by the Aspergillus fumigatus AtrR factor requires a conserved C-terminal domain
Source: mSphere. 2024 Jul 8;9(7):e00425-24. doi: 10.1128/msphere.00425-24 (PMC11288021; doi:10.1128/msphere.00425-24)
Supplement: Supplemental Figure and Tables — Figure S1; Tables S1 and S2. [file msphere.00425-24-s0001.pdf]

Supplemental data

Supplemental Figure 1. **Equivalent expression of alanine-scanning mutants**. A. Strains expressing the indicated alanine-scanning mutant forms of AtrR were analyzed by western blotting with the anti-AtrR antibody as described in the text. Molecular mass markers are indicated to the right side of each panel (M, Marker proteins). B. Levels of atrR mRNA for each strain were assessed using RT-qPCR. The level of each mRNA was normalized to that seen for the wild-type *atrR* gene grown in the absence of voriconazole.

Supplemental Figure 1. Ror, et al.

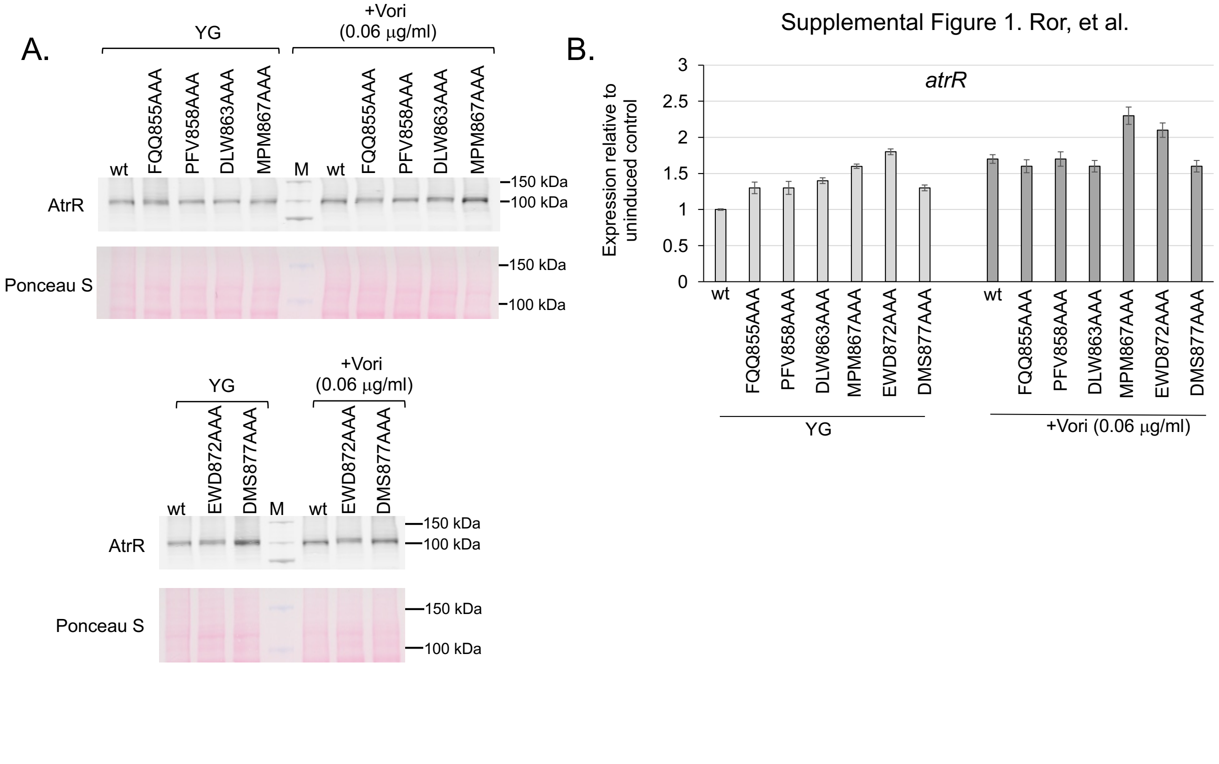

Strain Number.	Strain Name	Parent	Genotype	Reference
1	AfS35	D141	*akuAΔ::loxP*	FGSC
2	SRF59	AfS35	*atrR*::*ble*	This study
3	SRF60	AfS35	FQQ855AAA *atrR*::*ble*	This study
4	SRF61	AfS35	PFV858AAA *atrR*::*ble*	This study
5	SRF62	AfS35	DLW863AAA *atrR*::*ble*	This study
6	SRF63	AfS35	MPM867AAA *atrR*::*ble*	This study
7	SRF64	AfS35	EWD872AAA *atrR*::*ble*	This study
8	SRF65	AfS35	DMS877AAA *atrR*::*ble*	This study
9	SRF66	AfS35	Δ855-879 *atrR*::*ble*	This study
10	SRF67	AfS35	1-654 *atrR*::*ble*	This study
11	SRF68	AfS35	1-754 *atrR*::*ble*	This study
12	SRF69	AfS35	1-854 *atrR*::*ble*	This study
13	SRF70	AfS35	Δ655-754 *atrR*::*ble*	This study
14	SRF71	AfS35	Δ655-854 *atrR*::*ble*	This study
15	SPF323	AfS35	*atrRΔ::hph*	This study

Supplemental table 1. **Strains used in this work**.

Vector	Parent	Description
Control Clone		
pSR28	pUC19	*atrR* (198bp - C-terminal with stop codon) +*cyc1* terminator+PhleoR+atrR^{term} (45bp after *atrR* stop codon)
Internal Deletion/C-terminal deletion Clones		
pSR30 (Δ855-879 *atrR*)	pSR28	*atrR* (270bp-C-terminal with stop codon 855-879Δ)+Cyc1^{term}+Phleo^{R}+atrR^{term} (45bp after *atrR* stop codon)
pSR37 (1-654 *atrR*)	pSR28	*atrR* (95bp ahead of 655aa+stopcodon) +Cyc1^{term}+Phleo^{R}+atrR^{term} (45bp after *atrR* stop codon)
pSR38 (1-754 *atrR*)	pSR28	*atrR* (105bp ahead of 655aa+stopcodon) +Cyc1^{term}+Phleo^{R}+atrR^{term} (45bp after *atrR* stop codon)
pSR39 (1-854 *atrR*)	pSR28	*atrR* (219bp ahead of 855aa+stopcodon) +Cyc1^{term}+Phleo^{R}+atrR^{term} (45bp after *atrR* stop codon)
pSR40 (Δ655-754 *atrR*)	pSR28	*atrR* (518bp downstream of stop codon with amino acid 655 to 754 deleted) +

		$Cyc1^{term}$+$Phleo^{R}$+$atrR^{term}$ (45bp after *atrR* stop codon)
pSR41 (Δ655-854 *atrR*)	pSR28	*atrR* (218bp downstream of stop codon with amino acid 655 to 854 deleted) + $Cyc1^{term}$+$Phleo^{R}$+$atrR^{term}$ (45bp after *atrR* stop codon)
Alanine Mutagenesis Clones		
pSR31 (FQQ855AAA *atrR*)	pSR28	TTTCAACAG substituted with gcggcggcG*
pSR32 (PFV858AAA *atrR*)	pSR28	CCCTTCGTC substituted with gCCgcgGcC*
pSR33 (DLW863AAA *atrR*)	pSR28	GATTTGTGG substituted with GccgcGGcG*
pSR34 (MPM867AAA *atrR*)	pSR28	ATGCCGATG substituted with gccgCGgcG*
pSR35 (EWD872AAA *atrR*)	pSR28	GAATGGGAC substituted with GccgcgGcC*
pSR36 (DMS855AAA *atrR*)	pSR28	GACATGTCA substituted with GcCgcGgCA*

*Small letters indicate the changed nucleotides leading to the triple alanine substitution.

Supplemental table 2. **Plasmids used in this work**.
